# Platelet Count Measured Prior to Cancer Development Is a Risk Factor for Future Symptomatic Venous Thromboembolism: The Tromsø Study

**DOI:** 10.1371/journal.pone.0092011

**Published:** 2014-03-18

**Authors:** Hilde Jensvoll, Kristine Blix, Sigrid K. Brækkan, John-Bjarne Hansen

**Affiliations:** Hematological Research Group, Division of Internal Medicine, Department of Clinical Medicine, University of Tromsø, University Hospital of North Norway, Tromsø, Norway; Albert Einstein College of Medicine, United States of America

## Abstract

**Background:**

Elevated platelet count is associated with risk of venous thromboembolism in cancer patients initiating chemotherapy. It is not known whether this risk by platelet count is causal or merely reflects the malignant disease. We investigated whether pre-cancer platelet count alone or together with high leukocyte count was associated with risk of venous thromboembolism in subjects who did and did not develop cancer during follow-up in a population-based cohort study.

**Methods:**

Platelet count and other baseline characteristics were measured in 25160 initially cancer-free subjects who participated in the Tromsø Study in 1994–1995. Incident cancer and symptomatic venous thromboembolism events were registered up to December 31^st^, 2009. Multivariable Cox regression models were used to calculate hazard ratio for venous thromboembolism across categories of platelet count (<40^th^, 40–80^th^, and >80^th^ percentile) with 95% confidence interval.

**Results:**

During follow-up, 2082 subjects were diagnosed with cancer. Platelet count was measured on average 8.3 years before the cancer diagnosis. There were 129 venous thromboembolism events in the cancer cohort (13.5 per 1000 person-years) and 377 in the non-cancer cohort (1.2 per 1000 person-years). In cancer patients, pre-cancer platelet count above the 80^th^ percentile (≥295×10^9^/L) was associated with a 2-fold higher risk of venous thromboembolism (Hazard ratio: 1.98, 95% confidence interval 1.21–3.23) compared to platelet count below the 40^th^ percentile (<235×10^9^/L). Concomitant high platelet and leukocyte counts showed a synergistic effect on the VTE risk. In cancer-free subjects, no association was found.

**Comment:**

In conclusion, pre-cancer platelet count was associated with risk of symptomatic venous thromboembolism in cancer patients, but not in cancer-free subjects. Our findings suggest that platelet count and platelet-leukocyte interactions may play a role in the pathogenesis of cancer-related venous thromboembolism.

## Introduction

The association between malignant disease and venous thromboembolism (VTE) was described by Armand Trousseau in the 1860s [1]. VTE, which includes deep venous thrombosis and pulmonary embolism, is still a frequent complication and a leading cause of death in cancer patients [2]. Overall, cancer is associated with 20–30% of the incident VTE cases [2]. A recent meta-analysis reported that the annual incidence of VTE in patients with cancer varied between 0.5% and 20%, depending on cancer sites, stage, cancer treatment and time since diagnosis [3]. Furthermore, cancer patients with VTE have more bleeding complications on anticoagulation treatment [4], higher rates of recurrent VTE [4] and more frequent and prolonged hospital stays [5] compared to VTE patients without malignancy.

Platelets are essential in hemostasis and the formation of both arterial [6] and venous thrombosis [7]._ENREF_12 Cancer represents a hypercoagulable state where activated platelets promote angiogenesis, tumor progression and metastasis [8], [9]. An elevated platelet count is a common finding and a strong predictor of decreased survival in cancer patients [8], [10]. Platelet count is not associated with future VTE in population-based cohorts [11]–[13], but studies of cancer patients initiating chemotherapy have demonstrated that a high platelet count predicts increased risk of VTE [14]–[16]. Since an elevated platelet count in patients with active cancer might merely reflect an aggressive disease state with a higher thrombotic potential, it is not known whether there is a causal relationship between platelet count and VTE risk in cancer patients.

To address this question, we used data from the Tromsø Study, a large population-based cohort study, to investigate whether pre-cancer platelet count was associated with increased risk of symptomatic VTE in subjects who developed cancer during follow-up and in subjects who remained cancer-free. Recently, high leukocyte count measured prior to cancer development was shown to predict VTE in cancer patients [17]. Together with a biological rationale for platelet-leukocyte interactions in venous thrombosis [7], [18], this encouraged us to examine the joint effect of platelet and leukocyte counts on future risk of VTE.

## Methods

### Ethics statement

The study was approved by the Regional Committee for Medical and Health Research Ethics in Northern Norway, and the participants gave their informed written consent.

### Study population

Participants were recruited from the fourth survey of the Tromsø Study, a single-center, prospective, population-based study which was carried out in 1994–1995. All inhabitants aged over 24 years living in the municipality of Tromsø were invited and 27 158 participated (77% of the eligible population). Subjects who did not consent to medical research (n = 202), who were no longer registered as inhabitants of the municipality of Tromsø (n = 44) at the time of enrollment, with a previous diagnosis of cancer (n = 764) or VTE (n = 53), or with missing values for platelet count (n = 808) were excluded. In order to minimize the possibility that platelet count at baseline could be confounded by occult malignancy, subjects with a cancer diagnosis during the first year after enrollment were excluded from the analyses (n = 127). Accordingly, the total study population consisted of 25 160 subjects. Incident cancer diagnosis and VTE events among the study participants were recorded from the date of enrollment (1994–95) to the end of follow up, December 31^st^, 2009.

### Baseline measurements

Baseline information in the Tromsø Study was obtained by physical examination, blood samples and self-administered questionnaires. The blood samples were collected from the antecubital vein and analyzed at the department of Clinical Chemistry, University Hospital of North Norway. For measurement of platelet counts, 5 ml of blood was drawn into a vacationer tube containing EDTA as an anticoagulant (K3- EDTA 40 μL, 0.37 mol/L per tube), and analyzed within 12 hours by an automated blood cell counter (Coulter Counter®, Coulter Electronics, Luton, UK). Body height and weight were measured in subjects wearing light clothing and no shoes. Body mass index was calculated as weight in kilograms, divided by the square of the height in meters (kg/m^2^). Information about current daily smoking status, history of cardiovascular disease (myocardial infarction, angina or stroke), diabetes, higher education (university/college level) and physical activity (exercise that caused sweating or breathlessness ≥ one hour per week) were obtained from the self-administered questionnaires.

### Identification and validation of cancer- and venous thromboembolism diagnosis

Information about the date of cancer diagnosis, location of the disease (ICD-7-codes 140-205) and cancer stage (localized disease or presence of regional/distant metastasis) was obtained from linkage to the Cancer Registry of Norway. Subjects with non-melanoma skin cancers (ICD 191.0–191.9) were classified as cancer-free. The Cancer Registry of Norway is a complete and valid registry; a recent evaluation of data quality displayed 98.8% completeness, of which 94% had organ specific morphology [19].

First life time VTE events during follow up were identified by searching the hospital discharge diagnosis registry, the radiology procedure registry and the autopsy registry at the University Hospital of North Norway, previously described by Braekkan et al [13]. The University Hospital of North Norway is the only hospital serving the municipality of Tromsø, and all relevant diagnostic procedures and hospital care are provided here. The discharge diagnosis registry includes both outpatient clinic visits and hospitalizations. The medical record for each potential case of VTE was reviewed by trained personnel, and a VTE event was considered verified and recorded when presence of clinical signs and symptoms of deep venous thrombosis or pulmonary embolism were combined with objective confirmation tests (compression ultrasonography, venography, spiral computed tomography, perfusion-ventilation scan, pulmonary angiography, autopsy), and resulted in a VTE diagnosis that required treatment. VTE cases from the autopsy registry were recorded when the death certificate indicated VTE as cause of death or a significant condition associated with death.

A VTE event was classified as cancer-related if it occurred within one year prior to the cancer diagnosis (occult cancer) or after (overt cancer). Provoking factors (other than cancer) at the time of VTE diagnosis were recorded for all VTE events. These included surgery or trauma within the previous 8 weeks, acute medical conditions (acute myocardial infarction, ischemic stroke or major infectious disease), immobilization (bed rest >3 days, wheel chair use, long haul travel exceeding 4 hours in the past 14 days) or other provoking factors described by the physician in the medical record (e.g. intravascular catheter).

### Statistical analyses

For each participant, person-years of follow-up were accrued from the date of inclusion in 1994–95 and to the date of a VTE event, migration, death or to the end of the study period (December 31^st^, 2009), whichever came first. Subjects who developed cancer during follow-up contributed with person-years in the non-cancer cohort from the baseline inclusion date until the date one year prior to their cancer diagnosis. Thereafter they contributed with person-years in the cancer cohort (from the date one year prior to cancer diagnosis) to the end of follow-up. The switch of cancer status one year prior to cancer diagnosis was chosen in order to include VTEs that occurred in the presence of occult cancer as cancer-related VTEs.

The STATA nptrend command, a nonparametric test for trend across ordered groups, was used to assess p-values for linear trends in distribution of baseline variables (Table 1) and cancer diagnoses (Table 2) across categories of platelet count. Multivariable Cox proportional hazards regression models were used to calculate hazard ratios (HRs) with 95% confidence intervals (CIs) for VTE across categories of platelet count (<40^th^, 40–80^th^, and >80^th^ percentiles), in the cancer and non-cancer cohorts respectively. The lower 40^th^ percentile was used as reference group. The first regression model (Model 1) was adjusted for age and sex, whereas the second model (Model 2) included age, sex, body mass index, smoking, mean platelet volume and leukocyte count as covariates. Among the baseline variables, only age, body mass index and leukocyte count were significantly associated with VTE in our analyses. However, sex, smoking and mean platelet volume were also included as covariates because of their highly significant trends across platelet count categories combined with their possible association with VTE [13], [20], [21]. The third Cox model (Model 3) was performed in cancer subjects only, and was an extended version of Model 2 which additionally included the variable cancer stage (localized or disseminated disease). Finally, the association between platelet count (continuous variable) and risk of VTE in subjects with and without cancer was visualized by generalized additive regression plots. Platelet count (log transformed) in these plots was modeled with a 4-degrees of freedom smoothing spline fit in Cox proportional hazard models including the variables in Model 2 as described above.

**Table 1:** Baseline characteristics across categories of platelet count in subjects who developed cancer and subjects who remained cancer-free during follow up; The Tromsø Study 1994–2009.

	Categories of platelet count (10^9^/L)			P for trend
	<235	235–294	≥295	
**Cancer**				
Subjects, n	906	773	403	
Age (years), mean ±1 SD	59.2±12.9	56.7±12.8	54.8±12.5	<0.001
Sex (females), % (n)	41.5 (376)	53.0 (410)	56.8 (229)	<0.001
BMI, mean ±1 SD	26.0±4.0	25.6±3.9	25.3±4.3	<0.001
Daily smoking, % (n)	37.5 (339)	41.4 (320)	49.1 (198)	<0.001
Physical activity*, % (n)	21.8 (195)	23.6 (181)	21.5 (86)	0.9
Self-reported DM, % (n)	4.4 (40)	2.9 (22)	0.7 (3)	<0.001
Self-reported CVD, % (n)	13.7 (124)	7.8 (60)	10.9 (44)	0.02
Higher education, % (n)	21.2 (190)	19.9 (154)	15.7 (63)	0.03
Leuk count, mean ±1 SD	6.7±2.5	7.3±1.9	8.1±2.1	<0.001
MPV, mean ±1 SD	9.2±1.0	8.6±0.7	8.2±0.7	<0.001
**Non-cancer**				
Subjects (n)	9055	9263	4760	
Age (years), mean ±1 SD	46.6±15.3	44.9±14.1	43.6±13.3	<0.001
Sex (female), % (n)	44.7 (4044)	54.7 (5065)	64.2 (3058)	<0.001
BMI, mean ±1 SD	25.0±3.6	25.1±3.8	25.3±4.1	0.001
Daily smoking, % (n)	33.2 (3004)	38.0 (3515)	42.0 (1994)	<0.001
Physical activity*, % (n)	32.8 (2949)	32.6 (3001)	27.2 (1286)	<0.001
Self-reported DM, % (n)	1.9 (170)	1.4 (129)	1.3 (62)	0.004
Self-reported CVD, % (n)	7.4 (669)	5.2 (479)	4.0 (190)	<0.001
Higher education, % (n)	33.3 (3009)	30.7 (2829)	27.2 (1293)	<0.001
Leuk count, mean ±1 SD	6.5±1.7	7.2±1.9	8.0±2.1	<0.001
MPV, mean ±1 SD	9.1±1.0	8.6±0.8	8.2±0.7	<0.001

*Physical activity ≥ °ne hour per week of activity that caused sweating or breathlessness.

Abbreviations: SD; standard deviation, BMI; Body mass index (kg/m^2^), DM; Diabetes mellitus, CVD; Cardiovascular disease, Leuk count; Leukocyte count (10^9^/L), MPV; Mean platelet volume (fL).

**Table 2:** Characteristics across categories of platelet count at the time of cancer diagnosis; The Tromsø Study 1994–2009.

	Categories of platelet count (10^9^/L)			P for trend
	<235	235–294	≥295	
Subjects, n	906	773	403	
Age (years), mean ±1 SD	68.1±12.5	65.7±12.4	63.7±12.3	<0.001
**Cancer stage**				
Local, % (n)	36.2 (328)	33.9 (262)	29.8 (120)	0.03
Regional, % (n)	21.0 (190)	20.2 (156)	25.8 (104)	0.1
Distant metastasis, % (n)	17.8 (161)	18.0 (139)	18.9 (76)	0.7
Unknown, % (n)	25.1 (227)	27.9 (216)	26.6 (103)	0.6
**Cancer site**				
Colon/rectum, % (n)	15.9 (144)	15.0 (116)	13.9 (56)	0.4
Prostate, % (n)	18.7 (169)	11.9 (92)	11.4 (46)	<0.001
Lung, % (n)	11.1 (101)	12.9 (100)	14.9 (60)	0.05
Breast, % (n)	11.3 (102)	13.8 (107)	12.2 (49)	0.4
Bladder and urinary tracts, % (n)	7.1 (64)	8.4 (65)	7.2 (29)	0.7
Hematopoietic/Lymphatic, % (n)	7.2 (65)	7.4 (57)	7.9 (32)	0.6
Gynecological, % (n)	6.2 (56)	7.2 (56)	7.2 (29)	0.4
Upper gastrointestinal*, % (n)	6.8 (62)	6.3 (49)	6.7 (27)	0.8
Central nervous system, % (n)	4.5 (41)	3.1 (24)	5.0 (20)	0.9
Pancreas, % (n)	4.1 (37)	2.8 (22)	2.0 (8)	0.04
All other sites, % (n)	7.2 (65)	11.0 (85)	11.7 (47)	0.003

*Includes es°phagus, stomach, small intestine, liver, gallbladder and biliary tract.

SD; standard deviation.

Statistical interactions between platelet count and the other variables in the models were tested by including cross-product terms in the proportional hazard models, and no interactions were found. However, a statistical interaction between platelet count and cancer was confirmed, which justified separate analyses for cancer and non-cancer subjects. The proportional hazards assumption was confirmed by an evaluation of the parallelism between the curves of the log-log survivor function.

For further elucidation of causality, we additionally investigated whether a high platelet count in itself was associated with cancer, using a Cox-regression model where person-years were counted from baseline inclusion until the date of cancer diagnosis (event), death (censored) or end of study (December 31, 2009), whichever came first.

Furthermore, we assessed the joint effect of platelet and leukocyte counts on VTE-risk. Four categories were generated by combining platelet count with leukocyte count: low-low: platelet count <295×10^9^/L and leukocyte count <8.6×10^9^/L, low-high: platelet count <295×10^9^/L and leukocyte count ≥8.6×10^9^/L, high-low: platelet count≥295×10^9^/L and leukocyte count <8.6×10^9^/L and high-high: platelet count≥295×10^9^/L and leukocyte count ≥8.6×10^9^/L. The low-low category was used as reference group in the Cox models. The simple regression model included sex and age, while the two more complex analyses were adjusted for age, sex, smoking, body mass index, mean platelet volume and stage. Rothman synergy index [22] was used to determine whether the joint effect from high leukocyte count and high platelet count on VTE risk exceeded the sum of the effect from each factor alone. Synergy index  =  (RR_ab_ -1)/(RR_a_ + RR_b_ -2). RR_ab_ is the relative risk of the joint exposure group; RR_a_ and RR_b_ are relative risk for the exposure of high leukocyte and high platelet count, respectively. A value above 1.0 suggests that effect of the joint exposures of two risk factors is greater than the sum of the separate effects. The proportion attributable to the interaction was calculated (AP =  (RRab-RRa-RRb+1)/RRab) to demonstrate the proportion of cases that could be explained by the interaction.

Statistical analyses were carried out using SPSS (version 19.0; IBM SPSS Statistics), STATA (version 13; Stata Corporation, College Station, TX, USA) and R (version 2.15.1 for windows).

## Results

In total, 2082 participants developed cancer during follow-up. Baseline characteristics in the cancer and the non-cancer cohort across categories of platelet count are presented in Table 1. The mean age in the cancer cohort (57 years) was higher than in the non-cancer cohort (45 years), and declined across categories of platelet count in both cohorts. Body mass index was slightly higher with higher platelet count only in the cancer-free cohort. The leukocyte count and the proportion of females and current smokers increased across platelet categories in both cohorts.

In subjects who developed cancer during follow-up, the mean time from baseline to cancer diagnosis was 8.3 years. Platelet count was not associated with risk of cancer (HR Model 2: 1.03, 95% CI 0.89–1.18) (Table S1). Cancer characteristics by increasing platelet count at the time of cancer diagnosis are shown in Table 2. The proportion of subjects with localized disease decreased across categories of platelet count. There were more subjects with prostate cancer in the lowest category of platelet count, which may be due to the higher age in this category. The proportion of subjects with lung cancer increased across categories, which was probably related to the effect of smoking on platelet count. The proportion of subjects with pancreas cancer decreased across categories.

The characteristics of cancer patients and non-cancer patients at the time of the symptomatic VTE event are presented in Table S2. There were 129 incident VTE events in the cancer cohort and 377 in the non-cancer cohort. In the cancer cohort, 19% of the subjects had VTE within one year prior to the cancer diagnosis, and 81% after. The mean age, the gender distribution, and the total proportion of provoking factors were essentially similar in the two cohorts.

**Figure 1:**
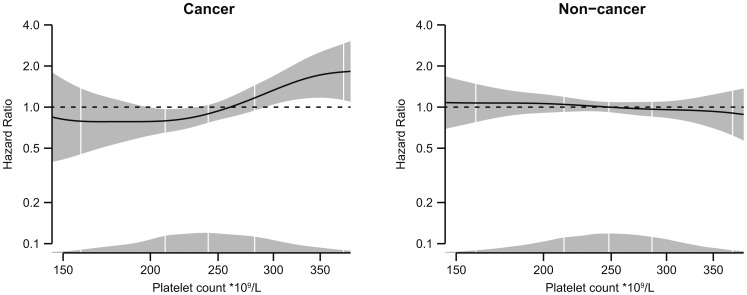
Platelet count and risk of symptomatic venous thromboembolism. Dose-response relationship between platelet count and risk of venous thromboembolism in cancer and non-cancer subjects obtained by generalized linear regression. The regression models are adjusted for age, sex, body mass index, smoking, leukocyte count and mean platelet volume. The solid lines show hazard ratios and the shaded areas represent 95% confidence intervals. Density plots show the distribution of platelet count, and white vertical lines indicate 2.5^th^, 25^th^, 50^th^, 75^th^ and 97.5^th^ percentiles.

Incidence rates and hazard ratios for VTE across categories of platelet count are shown in Table 3. The mean observational time in the cancer-cohort was 4.6 years versus 12.3 years in the non-cancer cohort. The overall crude incidence of VTE was 13.5 per 1000 person-years in the cancer-cohort and 1.2 per 1000 person years in the non-cancer cohort. In cancer patients, a 2-fold increased risk of VTE was found by pre-cancer platelet count above the 80^th^ percentile (≥295×10^9^/L) compared with platelet count below the 40^th^ percentile (<235×10^9^/L) both in the age-and sex-adjusted model (HR 1.97, 95% CI 1.27–3.06) and in the multivariable model (Model 2) (HR 1.98, 95% CI 1.21–3.23.) Further adjustment for cancer stage (Model 3) did not change the risk estimate (HR 1.93, 95% CI 1.18–3.16). Platelet count was not associated with VTE in the non-cancer cohort (HR Model 2: 0.95, 95% CI 0.69–1.32), and separate analyses for provoked and unprovoked VTE showed similar risk estimates (Table S3). When platelet count was modeled as a continuous variable, a clear dose-response relationship between increasing platelet count and risk of VTE was observed in the cancer cohort, whereas no relationship was found in the non-cancer cohort (Figure 1).

**Table 3:** Incidence rates (IRs) and hazard ratios (HRs) for symptomatic venous thromboembolism by increasing platelet count with 95% confidence intervals; The Tromsø Study 1994–2009.

Platelet count*	PY†	Events	IR‡	HR Model 1	HR Model 2	HR Model 3
**Cancer**						
<235	4222	46	10.9 (8.2–14.5)	Ref	Ref	Ref
235–294	3511	46	13.1 (9.8–17.5)	1.22 (0.81–1.84)	1.24 (0.80–1.90)	1.22 (0.79–1.88)
≥295	1806	37	20.5 (14.8–28.3)	1.97 (1.27–3.06)	1.98 (1.21–3.23)	1.93 (1.18–3.16)
*P for trend*				*0.004*	*0.008*	*0.01*
**Non-cancer**						
<235	120223	173	1.4 (1.2–1.7)	Ref	Ref	-
235–294	124179	137	1.1 (0.9–1.3)	0.89 (0.71–1.11)	0.89 (0.70–1.14)	-
≥295	64162	67	1.0 (0.8–1.3)	0.95 (0.71–1.26)	0.95 (0.69–1.32)	-
*P for trend*				*0.5*	*0.6*	*-*

*10^9^/L.

† Person years. Subjects who develop cancer during follow-up are treated as non-cancer subjects until one year prior to the cancer diagnosis.

‡ Incidence per 1000 person years.

Model 1: Adjusted for age and sex.

Model 2: Adjusted for age, sex, body mass index, smoking, leukocyte count and mean platelet volume.

Model 3: Model 2+ cancer stage (defined as localized or disseminated disease).

The joint effects of platelet count and leukocyte count on VTE risk are shown in Table 4. Cancer patients in the high-high category (platelet count ≥295×10^9^/L and leukocyte count ≥8.6×10^9^/L) had an age-and sex-adjusted 3-fold higher risk of VTE (HR: 3.00, 95% CI 1.80–5.00) compared with the low-low category (platelet count <295×10^9^/L and leukocyte count<8.6×10^9^/L). In the multivariable analysis (Model 2), HR was 3.09 (95% CI 1.80–5.32) in the upper category compared with the lowest category. Additional adjustment for stage did not affect the result (HR: 2.96, 95% CI1.72–5.08). A synergy index of 2.25 (calculated from the risk estimates adjusted for the variables in Model 2) suggested a synergistic effect, and the proportion attributable to the interaction (AP) was 38%. In the non-cancer cohort there was no association between the various combinations of platelet and leukocyte count and risk of VTE.

**Table 4:** Incidence rates (IRs) and hazard ratios (HRs) for symptomatic venous thromboembolism by categories of platelet count and leukocyte count with 95% confidence intervals; The Tromsø Study 1994–2009.

Platelet count*	Leukocyte count*	PY†	Events	IR‡	HR Model 1	HR Model 2	HR Model 3
**Cancer**							
<295	<8.6	6513	72	11.1 (8.8–13.9)	Ref	Ref	Ref
	≥8.6	1220	20	16.4 (10.6–25.4)	1.49(0.91–2.46)	1.46 (0.87–2.44)	1.45 (0.87–2.43)
≥295	<8.6	1221	18	14.7 (9.3–23.4)	1.41(0.84–2.36)	1.47 (0.86–2.51)	1.45 (0.85–2.49)
	≥8.6	585	19	32.5 (20.7–50.9)	3.00(1.80–5.00)	3.09 (1.80–5.32)	2.96 (1.72–5.08)
**Non-cancer**							
<295	<8.6	203423	272	1.3 (1.2–1.5)	Ref	Ref	-
	≥8.6	40904	38	0.9 (0.7–1.3)	0.90 (0.64–1.27)	0.87 (0.61–1.24)	-
≥295	<8.6	42521	41	1.0 (0.7–1.3)	0.87 (0.63–1.22)	0.89 (0.64–1.26)	-
	≥8.6	21626	26	1.2 (0.8–1.8)	1.25 (0.84–1–88)	1.31 (0.86–1.99)	-

*10^9^/L.

† Person years. Subjects who develop cancer during follow-up are treated as non-cancer subjects until one year prior to the cancer diagnosis.

‡ Incidence rate per 1000 person years.

Model 1: Adjusted for age and sex.

Model 2: Adjusted for age, sex, smoking, body mass index and mean platelet volume.

Model 3: Model 2+ cancer stage (defined as localized or disseminated disease).

## Discussion

Our population-based study is, to the best of our knowledge, the first to identify platelet count prior to cancer development as a risk factor for symptomatic VTE in cancer patients. Subjects with pre-cancer platelet count above the 80^th^ percentile (≥295×10^9^/L) had a 2-fold increased VTE risk compared to those below the 40^th^ percentile (<235×10^9^/L). The combination of platelet and leukocyte counts in the upper quintiles had a synergistic effect and yielded a 3-fold increased risk of VTE. In contrast, neither high platelet count alone nor the combined effect of high platelet and high leukocyte count did influence the risk of VTE in subjects who remained cancer-free.

The platelet count is determined by both inherited [23], [24] and environmental factors [25]. Platelets play a central role in hemostasis and thrombosis [6], [7], but in accordance with our findings in the non-cancer cohort, the platelet count has not been associated with future risk of VTE in the general population [11]–[13]. Conversely, a significant association between reactive thrombocytosis and VTE has been confirmed in hospitalized medical patients [26], intensive care unit patients [27] and trauma patients [28], [29]. There is also growing evidence that platelet count is predictive of VTE in cancer patients. Several studies have revealed that platelet count predicts VTE in cancer patients treated with chemotherapy [14]–[16], and ambulatory cancer patients with pre-chemotherapy platelet count ≥350×10^9^/L had a 2.8-fold increased risk of VTE [15]. Thrombocytosis prior to cancer surgery also predicts postoperative VTE [30].

Since thrombocytosis in cancer patients may reflect the inflammatory state associated with cancer, previous surgery or comorbid conditions (e.g. infections), these studies are not designed to evaluate the causality of platelet count in the development of cancer-related VTE. In our study, the platelet counts represent normal values in a general population, where the proportion of subjects with recent surgery or acute medical conditions is expected to be low. Furthermore, subjects with cancer before or within one year after the baseline measurement were excluded, and the participants developed cancer on average 8 years after baseline. It is therefore reasonable to assume that the number of individuals with platelet counts potentially influenced by occult cancer is negligible. This is further supported by the fact that platelet count was not associated with risk of cancer in our study. Thus, our findings indicate that elevated platelet count, predictive of VTE risk in cancer patients in previous studies [14]–[16], is not merely an innocent bystander reflecting the malignant disease. The VTE risk by platelet count may be mediated through high pre-cancer values instead of the rise in platelet count due to cancer. While high platelet counts in a general, healthy population apparently does not increase the risk of VTE, a malignant environment seem to convert high platelet count into a risk factor. Alternatively, our findings may be explained by the thrombosis potential model [31], suggesting that a high platelet count in combination with another strong risk factor, like cancer, is sufficient to reach the threshold for thrombosis.

Platelets promote cancer progression [8], [9], and an elevated platelet count is a common finding in cancer patients which is associated with decreased survival [8], [10]. As a consequence, pre-cancer platelet count could represent a common underlying risk factor for both cancer and cancer-related VTE, but in our study we did not find pre-cancer platelet count predictive of cancer development. Potentially, a high pre-cancer platelet count could be associated with more aggressive cancer development, for instance towards a more advanced cancer stage, which is a well-known risk factor for VTE [2]. However, there were no remarkable differences in the distribution of cancer sites across categories of platelet count, and adjustment for disseminated disease (regional/distant metastasis) at the time of cancer diagnosis did not weaken pre-cancer platelet count as a risk factor for VTE in our analyses.

The underlying mechanism by which platelet count may contribute to VTE in cancer patients is not clear. Activated platelets are suggested to be involved in chemotherapy-mediated risk of VTE [32]. Both activated platelets and platelet-derived microparticles provide a procoagulant membrane surface for the activation of thrombin [33], which again enhances platelet-tumor interaction and further tumor progression [34]. Thus, a high platelet count may result in a larger membrane surface which facilitates interaction with tumor cells and coagulation factors and thereby promote coagulation activation.

Platelet count ≥350×10^9^/L and leukocyte count ≥11×10^9^/L have been recognized as risk factors for VTE in a study of 4066 cancer patients prior to chemotherapy [35]. In the present study we demonstrated that the combination of high platelet and high leukocyte counts had synergistic effect on VTE risk, and that 38% of the VTE events could be attributed to the interaction. This provides epidemiological support for a biological interaction between platelets and leukocytes on development of VTE. A recent experimental study on the interaction between leukocytes and platelets in cancer-associated thrombosis further support a mutual enhancement of prothrombotic functions [18]. In a murine model using carcinoma mucins, the formation of platelet-rich microthrombi was dependent on bidirectional signaling via P-selectin on platelets and L-selectin and PSGL-1 on neutrophils. Cathepsin G, a potent platelet agonist secreted from neutrophils, was important in the formation of mucin-induced thrombosis [18]. P-selectin is expressed on activated platelets and interacts with cancer cells, endothelium and leukocytes [36], and the amount of leukocyte-platelet aggregates in the circulation is directly correlated with platelet expression of P-selectin [37]. P-selectin is also recognized as a risk factor for cancer-related venous thrombosis [38]. A study of VTE in mice (without cancer) found that platelet-leukocyte interaction was dependent on the platelet receptor GP1bα, and suggested that this interaction promoted leukocyte recruitment and release of neutrophil extracellular traps (NETs) from neutrophils [7]. These mechanisms support the observed synergistic effect of high platelet and leukocyte counts on VTE risk in cancer patients.

The main strengths of our study are the prospective design, the high participation rate and the well validated VTE events and cancer diagnoses. The size of the original cohort and the long-term follow-up made it possible to investigate VTE in cancer patients with respect to pre-cancer exposure. Such baseline measurements are not biased by cancer characteristics, a challenge for interpretations in many studies. Some limitations should also be mentioned. The cancer treatment modality was not available in our study, and the rather limited size of the cancer cohort restricted our possibilities to evaluate the impact of cancer sites on the association between platelet count and risk of VTE. Moreover, we cannot answer how platelet indices during active cancer are related to pre-cancer measurements. The platelet count is affected by several factors, and as in all cohort studies, unrecognized confounding cannot be ruled out. Lastly, the platelet count in our study was based on one single measurement. However, a review covering 316 studies reported that the within-subject biologic variation for platelet count is only 9.1% [39].

In conclusion, platelet count in the upper clinical range predicted VTE-risk in patients who developed cancer during follow-up, but not in subjects who remained cancer-free. Additionally, the combination of high platelet and high leukocyte counts had synergistic effect on risk of VTE in cancer patients. Our findings suggest that platelet count and platelet-leukocyte interactions may play a role in the pathogenesis of cancer-related VTE.

## Supporting Information

Table S1 **Incidence rates (IRs) and hazard ratios (HRs) for cancer by increasing platelet count with 95% confidence intervals; The Tromsø Study 1994–2009.**
(DOC)

Table S2 **Characteristics of cancer and non-cancer related symptomatic venous thromboembolism (VTE) at the time of VTE diagnosis; The Tromsø Study 1994–2009.**
(DOC)

Table S3 **Incidence rates (IRs) and hazard ratios (HRs) for provoked and unprovoked symptomatic venous thromboembolism by increasing platelet count in cancer-free subjects with 95% confidence intervals; The Tromsø Study 1994–2009.**
(DOC)
