# Supplementary material for: Platelet Count Measured Prior to Cancer Development Is a Risk Factor for Future Symptomatic Venous Thromboembolism: The Tromsø Study
Source: PLoS One. 2014 Mar 18;9(3):e92011. doi: 10.1371/journal.pone.0092011 (PMC3958406; doi:10.1371/journal.pone.0092011)
Supplement: Table S1 — Incidence rates (IRs) and hazard ratios (HRs) for cancer by increasing platelet count with 95% confidence intervals; The Tromsø Study 1994–2009. (DOC) [file pone.0092011.s001.doc]

**Table S1.** Incidence rates (IRs) and hazard ratios (HRs) for cancer by increasing platelet count with 95% confidence intervals; The Tromsø Study 1994-2009.

| **Platelet count*** | **PY†** | **Events** | **IR‡** | **HR Model 1** | **HR Model 2** |
| --- | --- | --- | --- | --- | --- |
| < 235 | 135118 | 906 | 6.7 (6.3-7.2) | Ref | Ref |
| 235 - 294 | 139292 | 773 | 5.5 (5.2-6.0) | 0.97 (0.88-1.07) | 0.94 (0.85-1.04) |
| ≥ 295 | 71980 | 403 | 5.6 (5.1-6.2) | 1.11 (0.98-1.25) | 1.03 (0.90-1.18) |
| *P for trend* |  |  |  | *0.2* | *0.9* |

*10^9^/L

†Person years

‡Incidence per 1000 person years.

Model 1: Adjusted for age and sex.

Model 2: Adjusted for age, sex, smoking, body mass index, leukocyte count and mean platelet volume.
