# Supplementary material for: Platelet Count Measured Prior to Cancer Development Is a Risk Factor for Future Symptomatic Venous Thromboembolism: The Tromsø Study
Source: PLoS One. 2014 Mar 18;9(3):e92011. doi: 10.1371/journal.pone.0092011 (PMC3958406; doi:10.1371/journal.pone.0092011)
Supplement: Table S2 — Characteristics of cancer and non-cancer related symptomatic venous thromboembolism (VTE) at the time of VTE diagnosis; The Tromsø Study 1994–2009. (DOC) [file pone.0092011.s002.doc]

**Table S2.** Characteristics of cancer and non-cancer related symptomatic venous thromboembolism (VTE) at the time of VTE diagnosis; The Tromsø Study 1994-2009.

|  | **Cancer-related VTE** | **Non-cancer VTE** | **P** |
| --- | --- | --- | --- |
| Subjects, n | 129 | 377 |  |
| Age (years), mean ± 1 SD | 68.2 ± 11.8 | 67.5 ± 14.4 | 0.6 |
| Sex (females), % (n) | 51.2 (66) | 53.8 (203) | 0.6 |
| **Provoking factors** | | | |
| Surgery*, % (n) | 14.7 (19) | 16.2 (61) | 0.7 |
| Trauma*, % (n) | 0.8 (1) | 8.0 (30) | 0.003 |
| Acute medical condition†, % (n) | 15.5 (20) | 14.9 (56) | 0.9 |
| Immobilization‡, % (n) | 22.5 (29) | 18.6 (70) | 0.3 |
| Other factor§, % (n) | 7.0 (9) | 3.2 (12) | 0.06 |
| Total provoked¶, % (n) | 45.7 (59) | 45.1 (170) | 0.9 |

*Within 8 weeks before the VTE event.

†Myocardial infarction, ischemic stroke or major infectious disease.

‡Bed rest > 3 days, wheelchair, long haul travel >4 hours in the past 14 days.

§Other provoking factor described by the physician, e.g. intravascular catheter.

¶Proportion of subjects with one or more of the provoking factors listed in the table.

SD; standard deviation
