# Supplementary material for: Platelet Count Measured Prior to Cancer Development Is a Risk Factor for Future Symptomatic Venous Thromboembolism: The Tromsø Study
Source: PLoS One. 2014 Mar 18;9(3):e92011. doi: 10.1371/journal.pone.0092011 (PMC3958406; doi:10.1371/journal.pone.0092011)
Supplement: Table S3 — Incidence rates (IRs) and hazard ratios (HRs) for provoked and unprovoked symptomatic venous thromboembolism by increasing platelet count in cancer-free subjects with 95% confidence intervals; The Tromsø Study 1994–2009. (DOC) [file pone.0092011.s003.doc]

**Table S3.** Incidence rates (IRs) and hazard ratios (HRs) for provoked and unprovoked symptomatic venous thromboembolism by increasing platelet count in cancer-free subjects with 95% confidence intervals; The Tromsø Study 1994-2009.

| **Platelet count*** | **PY†** | **Events** | **IR‡** | **HR Model 1** | **HR Model 2** |
| --- | --- | --- | --- | --- | --- |
| **Provoked** | | | | | |
| < 235 | 119485 | 82 | 0.07 (0.06-0.09) | Ref | Ref |
| 235-294 | 123486 | 59 | 0.05 (0.04-0.06) | 0.81 (0.58-1.14) | 0.80 (0.56-1.15) |
| ≥ 295 | 63851 | 29 | 0.05 (0.03-0.07) | 0.88 (0.57-1.35) | 0.87 (0.53-1.42) |
| *P for trend* |  |  |  | *0.4* | *0.4* |
| **Unprovoked** | | | | | |
| < 235 | 119557 | 91 | 0.08 (0.06-0.09) | Ref | Ref |
| 235-294 | 123725 | 78 | 0.06 (0.05-0.08) | 0.95 (0.70-1.29) | 0.97 (0.70-1.35) |
| ≥ 295 | 63913 | 38 | 0.06 (0.04-0.08) | 1.01 (0.68-1.48) | 1.03 (0.66-1.59) |
| *P for trend* |  |  |  | *0.9* | *0.9* |

*10^9^/L

†Person years

‡Incidence per 1000 person years.

Model 1: Adjusted for age and sex.

Model 2: Adjusted for age, sex, smoking, body mass index, leukocyte count and mean platelet volume.
